# Protection and Installation of FBG Strain Sensor in Deep Boreholes for Subsurface Faults Behavior Monitoring

**DOI:** 10.3390/s21155170

**Published:** 2021-07-30

**Authors:** Sang-Jin Choi, Kwon Gyu Park, Chan Park, Changhyun Lee

**Affiliations:** 1 Petroleum & Marine Division, Korea Institute of Geoscience and Mineral Resources, 124 Gwahak-ro, Yuseong-gu, Daejeon 34132, Korea; sang-jin@kigam.re.kr; 2 Geologic Environment Division, Korea Institute of Geoscience and Mineral Resources, 124 Gwahak-ro, Yuseong-gu, Daejeon 34132, Korea; chan@kigam.re.kr

**Keywords:** FBG strain sensor, fault behavior monitoring, borehole installation and completion, sensor protection measures, fiber reinforced plastic (FRP) forming

## Abstract

Fiber optic sensors are gradually replacing electrical sensors in geotechnical applications owing to their immunity to electrical interference, durability, and cost-effectiveness. However, additional protective measures are required to prevent loss of functionality due to damage to the sensors, cables, or connection parts (splices and/or connectors) during installation and completion processes in borehole applications. We introduce two cases of installing fiber Bragg grating (FBG) strain sensors in 1 km boreholes to monitor the behavior of deep subsurface faults. We present our fiber-reinforced plastic (FRP) forming schemes to protect sensors and splices. We also present uniaxial load test and post-completion monitoring results for assessing the effects and performance of the protective measures. The uniaxial load test and post-completion monitoring show that FBG sensors are well protected by FRP forming without significant impact on sensor performance itself and that they are successfully installed in deep boreholes. In addition to summarizing our learning from experiences, we also suggest several points for consideration to improve the applicability of FBG sensors in borehole environment of the geotechnical field.

## 1. Introduction

With the development of optical measuring and packaging technologies, the scope for the use of optical fiber sensors has been expanding to various application fields. In this regard, fiber Bragg grating (FBG) sensors and distributed temperature sensing (DTS) are being actively applied for slope stability monitoring in the geotechnical fields [1,2,3,4] and temperature monitoring in the geophysical fields [4,5,6,7,8,9], respectively. Recently, distributed acoustic sensing (DAS), among distributed optical fiber sensing (DOFS) technologies, has been actively investigated for seismic survey and passive monitoring, including earthquake and micro-seismic monitoring. The efforts to use the DOFS technology for the purpose of pressure and static strain monitoring are also increasing [10,11,12,13,14,15].

Although nodal electric strain gauges have been mainly used for ground deformation and strain monitoring [16,17,18], the use of optical fiber sensors has increased in the last decade because they offer several advantages over nodal strain gauges. For instance, they can be installed more economically in a high-temperature, high-pressure environment, are unaffected by electric noise, can cover longer distances and wider regions effectively owing to their lower signal loss over long transmission distances, and can be used for long periods without special maintenance.

Owing to these advantages, FBG sensors have been applied to monitor ground deformation in the form of multiple FBG sensor arrays [7,8] and borehole sensors installed by attaching them to the outside [19,20,21,22,23] or inside [24] of a casing, and then coupled with formation by cementing [25,26,27].

However, borehole environments are somewhat harsh for optical fiber sensors. Available sensors are often limited by borehole dimensions and target depth, and are easily exposed to damage during the installation process. In addition, retrieval of sensors for maintenance purposes is impossible after they are permanently installed by cementing. Therefore, it is imperative to develop and apply a methodology for selecting the optimal sensor suited to the installation method and conditions, as well as to prepare additional protection measures to prevent the loss of functionality owing to damage to the sensor or connection parts (cable, splice, and/or connector) during the installation process.

This study aims to investigate the future considerations, protection methodology, problems, countermeasures, and key improvements in applying optical fiber sensors on-site in geoscience and geotechnical fields using boreholes. To this end, we introduced two cases in which optical fiber strain sensors were installed into a 1 km deep borehole for monitoring the deep ground/fault behavior in 2020.

## 2. Methods

### 2.1. Monitoring Well Design and Sensor Selection

Successive occurrences of onshore earthquakes, such as the Gyeongju earthquake (12 September 2016, the moment magnitude (M_w_) = 5.8) and the Pohang earthquake (15 November 2017, M_w_ = 5.4) have become triggers that raise the necessity for continuous monitoring of the behavior of deep faults present in the southeastern part of Korea. To this end, the observation station construction project known as The Earth Login Leverage for Underground Signals (TELLUS) has been operational since 2018. 

The TELLUS project aims to construct six complex geophysical observatories centered around major fault zones of the southeastern part of Korea and, as of 2020, the construction of two observation stations (TENG and TEBD) has been completed. Each observation station monitors microearthquakes, strains, temperatures, pressures, underground water levels, and precision ground displacements using the global navigation satellite system (GNSS). These monitoring items were selected based on the results of existing studies [28,29,30,31,32,33,34,35,36]. Among these, strain monitoring is a key parameter of the TELLUS observatory to investigate tectonic motion by continuously monitoring the stress and deformation changes over time.

**Figure 1:**
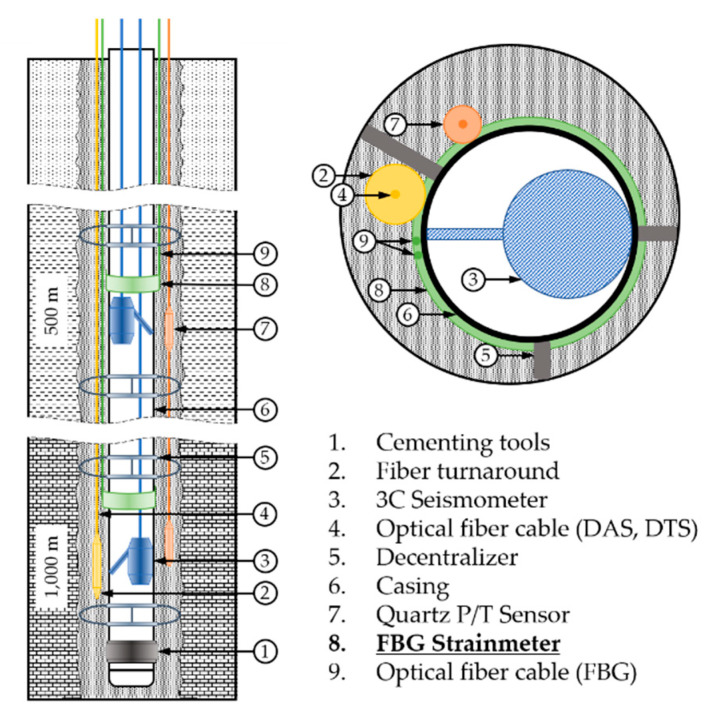
Monitoring borehole design at TELLUS project.

Other sensors, except GNSS, are installed inside the borehole at a depth of 1 km, and the schematic diagram of their arrangement inside the borehole is shown in Figure 1.

A 3-component (3C) seismometer is installed inside the casing to enable maintenance when necessary. The other sensors are installed by the tubing-convey method, i.e., attaching them to the exterior surface of a casing and running the casing into the borehole. The sensors are finally coupled permanently with formation by cementing. Based on the geological conditions, economic feasibility, and existing monitoring cases, the borehole diameter of the final section for sensor installation is determined to be 7 7/8 in (≅200 mm), and 4.5 in (≅114.3 mm), and the American Petroleum Institute (API) standard casing (grade: K-55; nominal weight T & C: 11.6 lbs/ft (≅17.26 kg/m); thickness: 0.25 in (≅6.35 mm); Lake Petro Co. Ltd., Dongying City, China) is selected for tubing.

The corresponding strain monitoring sensor selection conditions are summarized as follows: (1) the 9-component measurement for more accurately identifying the stress change can be conducted simultaneously at two different depths (500 m and 1000 m); (2) the stability and reliability of long-term monitoring with the permanent installation are secured; and (3) the installation is attachable to the outside of the casing with a diameter of 4.5 in (≅114.3 mm).

The sensor specifications that satisfy these conditions are listed in Table 1. The HBM Optical Rosette (OR, HBM, Darmstadt, Germany) sensor has three components arranged as a regular triangular-shaped sensor. The minimum attachable radius of curvature of the OR sensor is 25 mm, and three sensors can be installed on the surface of the casing with an outer diameter of 4.5 in (≅114.3 mm) at intervals of 120°. In other words, by installing three 3-axis OR sensors at one point at 120° intervals, a 9-component strain measurement is enabled. 

In addition to the OR sensor, a weldable strain sensor (WSS, HBM, Darmstadt, Germany), rugged temperature sensor (RTS, HBM, Darmstadt, Germany), and FBG acceleration sensor (FBG AC, FBG Korea, Daejeon, Korea) were installed. The WSS is for an auxiliary strain measurement, the RTS is for temperature correction of the FBG strain sensor, and the FBG AC is for determining the azimuthal direction of each FBG strain sensor component after the well completion. 

### 2.2. Sensor Casing Design

Six types of sensors are installed at one target depth (OR, WSS, RTS, FBG AC, Pressure/Temperature (P/T), and DTS/DAS) in the TELLUS monitoring boreholes. This is a high number, and the work needed to protect them is complicated and time-consuming. It is important to secure the integrity of the sensor installation/protection work by optimizing the arrangement of the sensors, control lines, and protection methods, as well as providing for a sufficient number of working hours. The sensor casing was pre-manufactured to ensure sufficient sensor protection working hours and enhance the efficiency of the installation work.

**Figure 2:**
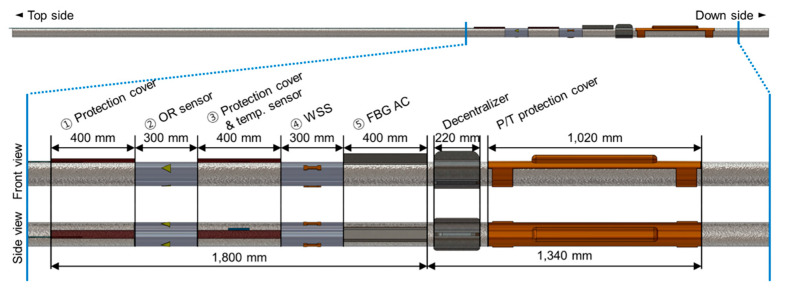
Front and side view of sensor casing design schematic.

**Figure 3:**
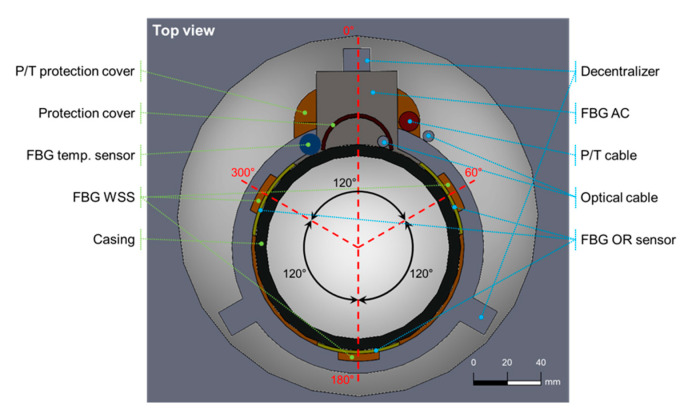
Top view schematic of sensor casing deployed inside borehole.

Figure 2 shows the placement of each sensor and protective devices in one casing. It consists of the FBG sensor part (➀–➄ in Figure 2), the decentralizer, and the P/T sensor, shown in this order from the upper part of the borehole. The fins of the decentralizer were asymmetrically designed and manufactured to protrude more than the attached sensors, and in this way prevent damage to the sensors by being stuck or swept onto the walls during sensor installation. The FBG sensor part comprises an optical fiber fusion splice protection area (➀ and ➂), strain sensors (➁ and ➃), and an acceleration sensor (➄). The strain sensors (OR and WSS) were attached to the strain sensor section (➁ and ➃) after removing the casing coating. The FBG strain sensors were installed at 60°, 180°, and 300° relative to the location of the longest fin of the decentralizer fixed to the casing (Figure 3). To protect the part in which the optical fiber was fusion-spliced (➀ and ➂ in Figure 2), a stainless steel pipe 400 mm long with a 40 mm diameter was cut into a half-moon shape and used for protection. Because the height of the protection areas and acceleration sensor are the longest among all installed sensors, they were arranged to be aligned to the highest decentralizer fin (0°) (Figure 3).

### 2.3. FBG Sensor Attachment and Protection Method

**Figure 4:**
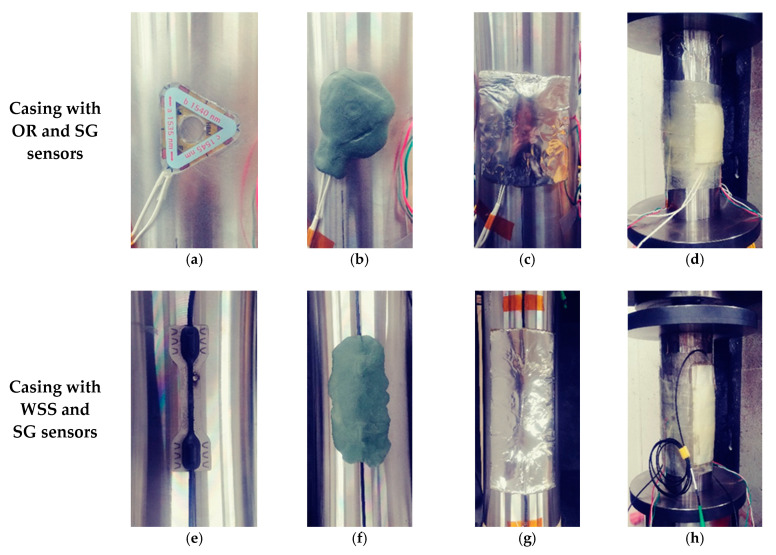
OR sensor and WSS mounting and protection steps: (**a**) OR sensor adhesion; (**b**) OR sensor with AK22; (**c**) OR sensor with ABM75; (**d**) OR sensor with FRP; (**e**) WSS adhesion; (**f**) WSS with AK22; (**g**) WSS with ABM75; (**h**) WSS with FRP.

If running the sensor casing down into the borehole without any protection, sensors and fiber connections may be damaged by being swept or caught on the borehole wall. Instantaneous overpressure and/or high-pressure cement flow may damage sensors and fiber connections during the cementing process. It is therefore critical to properly protect them in order to successfully install the FBG sensor into the borehole. An epoxy molding method using fiberglass, i.e., fiber-reinforced plastic (FRP) forming was therefore selected. In this process, a sticky putty that can be kneaded (AK22, HBM, Darmstadt, Germany) and a dough compound with a thickness of 3 mm (ABM75, HBM, Darmstadt, Germany) were used as intermediate protection material to prevent damage of sensors and fiber wiring from shock and overpressure delivered through the hard epoxy mold. Initially, the OR sensor and WSS were mounted to a grinded casing surface by using a Z70 (HBM, Darmstadt, Germany) adhesive (Figure 4a) and spot welding with a thermocouple welder (Figure 4e, HotSpot II, DCC Corporation, Pennsauken, NJ, USA), respectively. Each sensor was then protected with AK22 (Figure 4b,f) and ABM75 (Figure 4c,g). FBG sensors were finally protected with hand lay-up FRP (Figure 4d,h) forming by wrapping sensor sections with yarn-cloth fiberglass soaked with epoxy resin to form five layers. For the epoxy resin for FRP-forming, Resoltech Laminating Epoxy (resin 1050 and hardener 1058S, resoltech, Rousset, France), which can be cured at room temperature, has a high water-resistance, and can be quickly cured to reduce the on-site working hours, was used. 

### 2.4. Optical Fiber Cable

**Figure 5:**
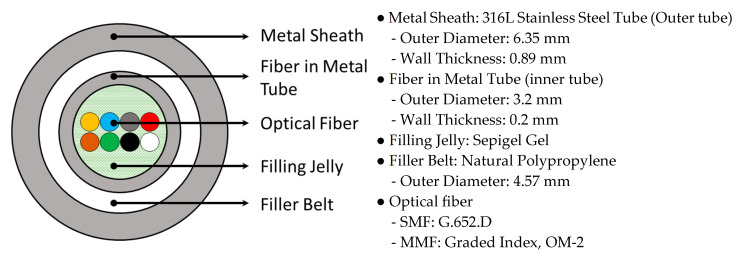
Optical fiber cable specifications.

The pigtail jacket of the FBG sensor, located at a depth of up to 1 km, is not durable enough to connect to the interrogator on the ground. To ensure the durability of the signal transmission cable, an optical fiber cable manufactured by the Prysmian Group (Farmington Hills, MI, USA), which can be used in harsh environments such as oil fields, was used (Figure 5). This cable has a tube-in-tube structure, each of which is made of 316L stainless steel to protect the inner optical fibers. The filler belt is located between two stainless steel tubes, and the inside of the inner tube (fiber in the metal tube) is filled with a gel to protect the optical fiber. 

Fiber optic cables can have a maximum of eight optical fiber cores; this composition can be selected during manufacturing. The FBG sensors installed at the TELLUS observatory (OR, WSS, RTS, FBG AC) use wavelengths of 1500–1600 nm and, hence, all cores can be composed of single-mode fibers (SMF). However, considering the case where DTS, which uses a multimode fiber (MMF), is required, an optical cable was constructed combining SMF and MMF (TENG observation station: SMF 4 EA+ MMF 4 EA; TEBD observation station: SMF 6 EA + MMF 2 EA).

Although the optical fiber cable used in the TENG observatory has four SMFs, only two SMFs are available for the FBG sensor connection because the others are used as Michelson interferometers for a future investigation on the possibility of nano-scale strain measurements [37,38]. The Michelson interferometers are installed at 90° and 270° positions in the casing shown in Figure 3. The length of the sensing arm of the interferometer is 3 m in the direction of the length of casing. If all FBG sensors at the same depth are connected in series, measurements can be performed with only one channel of the interrogator (FS22 SI, HBM, Darmstadt, Germany), which has a wavelength range of 100 nm and four channels. However, the OR sensor’s high loss and low reflectivity characteristics limit connecting sensors in a series; the sensors connected after an OR sensor may suffer from low SNR and large measurement error attributed to low light intensity reduced by the OR sensor. To reduce the measurement error by making the reflection intensity of each sensor similar to that of the other sensors, the light was separated using a 1 × 2 optical coupler and was located inside the protective cover (Figure 2 ➀). 

**Figure 6:**
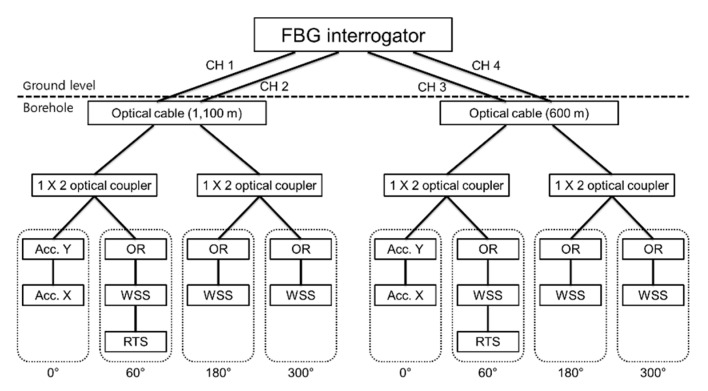
Fiber wiring diagram of the FBG interrogator, FBG sensor, and optical fiber cable at TENG observatory.

Figure 6 shows the wiring diagram of the optical fiber cable and FBG sensor from the FBG interrogator. The horizontal 2-axis (X and Y) acceleration sensors located at 0° relative to the casing are connected in series, and the OR sensors and WSS located at the same spot are also connected in series. The temperature sensor was connected to the first OR sensor in series. 

### 2.5. Optical Fiber Fusion Splicing

Optical fiber fusion splicing sections (Figure 2 ➀ and ➂), where the exterior jacket and the coating should be peeled off for splicing, are bound to vulnerable parts in terms of sensor protection. In particular, the fusion splice section between the sensor and the optical fiber cable (Figure 2 ➀) is the most vulnerable part, in terms of the integrity of the protective measures, because splicing and protection work should be quickly carried out on-site. Even if the sensor casing is manufactured in advance, it is inevitable to connect sensor parts and fiber optic cables on-site. This is because, when the optical cable is connected to the sensor in advance, the optical cable is wound around the casing in the process of rotating the sensor casing to connect it to the casing below. 

**Figure 7:**
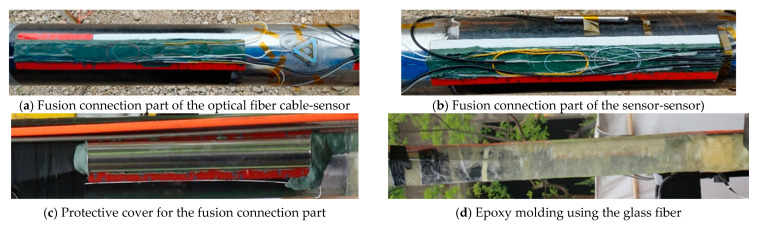
Protection method for the optical fiber fusion connection part: (**a**) Optical fiber cable-sensor fusion connection part (Figure 2 ➀); (**b**) Sensor-sensor fusion connection part (Figure 2 ➂); (**c**) Protective cover for the fusion connection part; (**d**) Epoxy molding using the glass fiber.

The damage that can occur at the optical fiber fusion splice includes (1) damage to the bare optical fiber coating due to the epoxy resin used for the epoxy molding (i.e., FRP-forming) [39]; (2) increase in micro-bending loss caused by the overlapping zone of the optical fiber being pressed excessively by the high pressure inside the borehole [40]; and (3) the damage to the optical fiber due to the height difference between the optical fiber and casing surface, as much of the wall thickness of the optical fiber cable. To reduce the possibility of such damage, all of the bases for each optical fiber crossover zone and optical fiber cable-casing drop-off zone were made of AK22, and the optical fiber fusion splice was arranged on the base (Figure 7a,b). To prevent damage due to the epoxy resin, a protective cover was applied, while the optical fiber fusion splice and the inner tubing part of the optical fiber cable were covered with AK22, and the empty space inside the protective cover was filled with AK22 (Figure 7c). This region was subjected to secondary protection with epoxy molding using glass fiber (Figure 7d). 

## 3. Results

### 3.1. Uniaxial Compression Test

Prior to the on-site application, the sensor casing for the laboratory test was manufactured using the same materials as the on-site materials (e.g., casing, sensor, and protection materials), and uniaxial compress tests were carried out. During the manufacturing of the samples, a strain gauge, widely used as an electrical resistive strain sensor was also attached to compare and evaluate the performance of the sensor, the effect of the installation method, and the protection measures.

**Figure 8:**
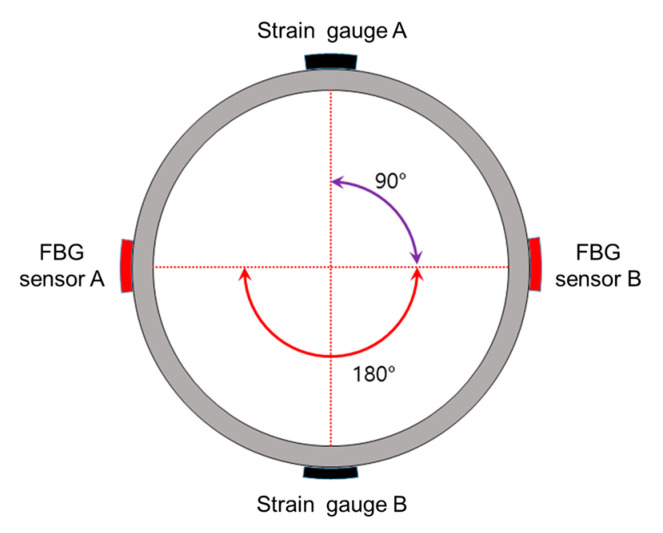
Cross-sectional diagram of the sensor casing and the sensor position.

Figure 8 shows a cross-sectional diagram of the test model, and a pair of FBG sensors and strain gauges (SGs, KFGS-5-120-D17-11, KYOWA, Tokyo, Japan) placed at right angles to each other. For the uniaxial compression test, an MTS 815 (315.01 load frame model, MTS, Eden Prairie, MN, USA) with a maximum load of 1600 kN (approximately 160 t) was used. The length of the sample (sensor casing) was manufactured to be 320 mm to meet the specifications of the MTS 815. Two casings were manufactured with OR-SG and WSS-SG sensor combinations (Figure 4). The sensors were located at the center of the casing height, and the SG sensor was attached using the CC-33A (KYOWA, Tokyo, Japan) adhesive, while the OR sensor and WSS were attached using the method described in Figure 4.

**Figure 9:**
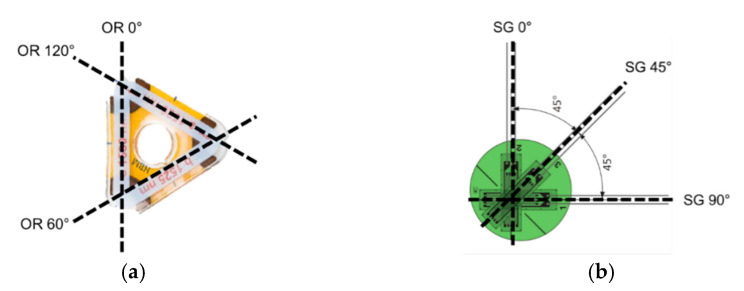
Side view of 3-axis sensor deployment: (**a**) OR sensor; (**b**) SG sensor.

The WSS-SG sample was attached in the direction vertical to the casing (loading direction) and the OR-SG sample, which is the 3-axis sensor, was arranged such that the 1-axis direction of each sensor was aligned with the load application direction (0°), as shown in Figure 9. 

For each of the two samples, a total of eight tests were carried out, including three before FRP-forming and one after. The initial load was set to 15 kN for the close contact of the MTS plate and the sample, and the strain was measured by increasing the load up to 588 kN. The FBG strain was measured using an FAZT I4Z (Femto sensing, Atlanta, GA, USA) interrogator, and the SG strain was measured using a dynamic strain amplifier (DPM-711B, KYOWA, Tokyo, Japan).

**Figure 10:**
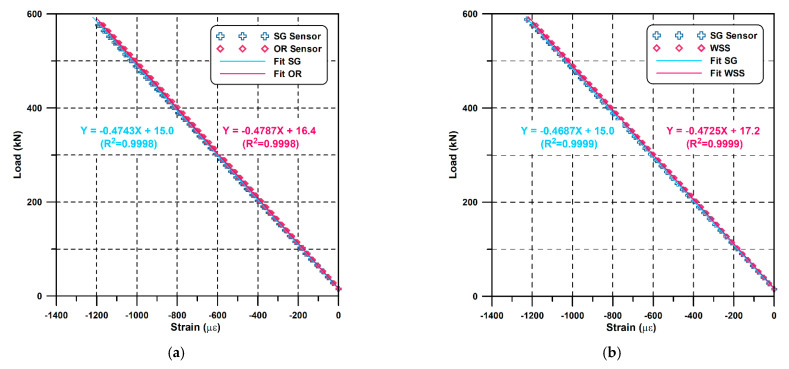
Uniaxial load test results before the FRP protection: (**a**) OR-SG; (**b**) WSS-SG.

The results before the FRP protection are shown in Figure 10. Each figure represents all the average values of each pair of sensors facing each other during three tests, repeated by rotating the casing by 45° to eliminate eccentric effects. As shown in the figure, in the load range of 15~588 kN, the R^2^ varies between 0.9998 and 0.9999, which indicates a high linearity of all sensors. Furthermore, the slope of the OR sensor and WSS differ only by 0.92% (0.0044 kN/με) and 0.81% (0.0038 kN/με) from the reference data (i.e., that of the SG sensor), respectively. Considering such a small error of less than 1 %, we could conclude that the OR sensor and WSS provide a reliable performance similar to the SG sensor.

**Figure 11:**
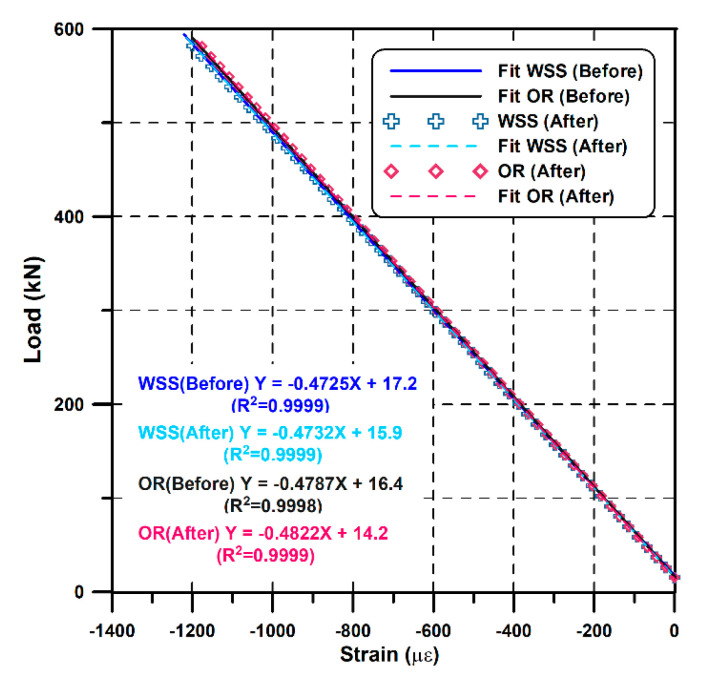
Comparison between the test before and after the FRP protection.

The effect of FRP-forming on the sensor response is presented in Figure 11, which shows the results before and after the protection of the OR sensor and WSS. The slope of the WSS and OR sensor differ only by 0.0007 kN/με and 0.00035 kN/με before and after the protection, with a high fidelity of R^2^ values of 0.9998 and 0.9999, respectively, which indicates that sensor protection does not affect the sensor response characteristics.

### 3.2. Issues and Countermeasures for the On-Site Installation

#### 3.2.1. Optical Fiber Cable Slip

**Figure 12:**
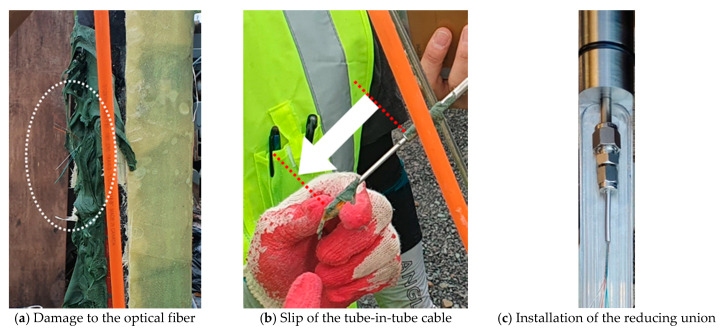
The damage to the optical fiber attributed to the slip of the tube-in-tube optical fiber cable: (**a**) Damage to the optical fiber; (**b**) Slip of the tube-in-tube optical fiber cable; (**c**) The optical fiber cable with the reducing union installed.

The first damage to the optical fiber of the manufactured sensor casing occurred when the sensor casing reached a depth of approximately 200 m at the TENG observatory. A retrieval investigation revealed that the optical fibers inside the fusion splicing section between the optical fiber cable and the sensor (Figure 2 ➀) were all cut off due to the slip phenomenon of the inner tube of the tube-in-tube structured optical fiber cable (an ellipse in Figure 12a,b). This tube-in-tube structure fiber optic cable that was wound on a reel before installation must be straightened during installation. Therefore, a 1/8 in (≅3.18 mm) inner tube with a smaller radius of curvature than a 1/4 in (≅6.35 mm) outer tube is forced to protrude further out of the outer tube during straightening, as shown in Figure 12b. As a part of the on-site measures for slip prevention, the reducing union (1/4 in (≅6.35 mm) to 1/8 in (≅3.18 mm)) was additionally installed at the end of the cable (Figure 10c). This is a method of holding the inner and outer tubes with 1/8 in (≅3.18 mm) and 1/4 in (≅6.35 mm) fittings, respectively, and inhibiting the tube from being pushed out by connecting these two with the reducing union. At least for the two cases in this study, this method was found to be effective in preventing cable slip. Controlled bending is a general method for preventing the slip of the tube-in-tube structure. However, this method is difficult to use in cases where excessive bending loss is expected because of the small borehole annulus and casing diameter, like the TELLUS borehole.

#### 3.2.2. Putty Deformation

**Figure 13:**
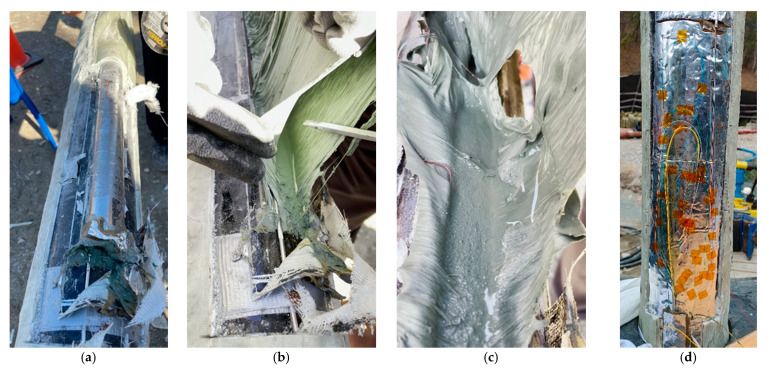
Identification of the reason for the optical fiber damage of the optical fiber fusion connection part and the changed optical fiber fusion connection part; (**a**) FRP removal; (**b**) Protective cover removal; (**c**) Optical fiber damage due to the AK22 deformation; (**d**) Optical fiber and fusion connection part placed on top of the ABM75.

Figure 13 shows the optical fiber damage that occurred during the construction of the TEBD observation station, which was the second observation station. Visual observation after removing the protective part of the optical fiber fusion splice (Figure 2 ➀) revealed that the AK22 putty that filled the inside of the protective cover was deformed by water which had infiltrated into the empty space inside the AK22 putty and protective cover (Figure 13).

This shows the importance of waterproofing performance in FRP protection. Optical sensors and optical fibers themselves are unaffected by water. However, this case shows that water infiltration into the protective measure due to the failure in waterproofing causes the deformation of the putty used as buffer material, and hence results in the bending and/or cutting off of the optical fiber.

The sensor casing of the second observatory (TEBD) presented in Figure 13 was manufactured using the bagging method, while the hand lay-up method was used at the first observatory (TENG). The bagging method has a more complex manufacturing process and is more time-consuming. However, it ensures superior physical properties (water tightness, interlayer adhesivity, and tensile/impulse strength) and can also reduce the FRP volume [41,42,43]. Nevertheless, the failure in waterproofing, as presented in Figure 13, is attributed to bubbles included in the epoxy resin during the excessive epoxy removal process and fingering-shaped channels formed while air flowed into the package.

To prevent putty deformation, which is the direct reason for the optical fiber damage due to water infiltration, the AK22 putty used as a base of fusion connection parts was substituted with a high elasticity ABM75 covering compound. After attaching ABM75 to the casing and fixing the optical fiber with Kapton tape (Figure 13d), the optical fiber was completely sealed using ABM75 once again. Finally, considering that the sensor part protected with FRP showed no issues, the optical fiber fusion splice was protected with the hand lay-up type FRP work without installing the half-pipe protective cover that was judged to be another problem.

#### 3.2.3. Other Improvements

Some items were revised for TEBD to minimize the risk elements, even though they did not result in a severe problem when installed at TENG as summarized below.

At first, the epoxy resin hardener was changed. Although only 1050 was used as the Resoltech Laminating Epoxy Resin employed for the FRP work, two hardeners, 1058S and 1059S, which have different gel times and exothermic temperature characteristics [44], were selectively used depending on the given working time and weather conditions. When using the 1059S hardener, the optical fiber coating may become damaged due to exothermic temperature around 217 °C [44]. In fact, a case of damage that appears to be due to this reason had occurred at the TENG observatory. Therefore, only 1056S with a low exothermic temperature of 184 °C [44] was used in TEBD. 

Secondly, we changed the FBG sensor arrangement. Basically, the design of the sensor casing installed at the TEBD observatory was similar to that at the TENG observatory. In case of TEBD, however, the FBG strain sensor installation space of the FBG sensor part was reduced by 100 mm (Figure 2 ➁ and ➃), and the installation direction of the FBG strain sensor was changed by 60℃ (Figure 3). By making these adjustments, the sensor pigtail optical fiber could be more stably located at the optical fiber fusion splice (Figure 2 ➀ and ➂). 

**Figure 14:**
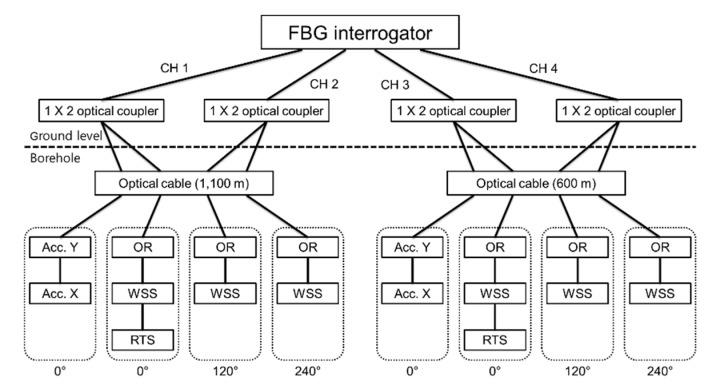
Connection method of the FBG interrogator, FBG sensor, and optical fiber cable at the TEBD observatory.

Thirdly, we changed the optical coupler location. The optical fiber cables used in the TENG observation station were SMF 4 EA+ MMF 4 EA. For the FBG measurement, only two SMFs were used, and a 1 × 2 optical coupler to control the FBG reflective light intensity had to be located inside the sensor casing protective cover (Figure 2 ➀). The optical fiber cables used in the TEBD observatory were SMF 6 EA + MMF 2 EA and four available SMFs. By changing the measurement wiring structure, as shown in Figure 14, the possibilities of damage were reduced by locating the optical coupler at ground level. 

**Figure 15:**
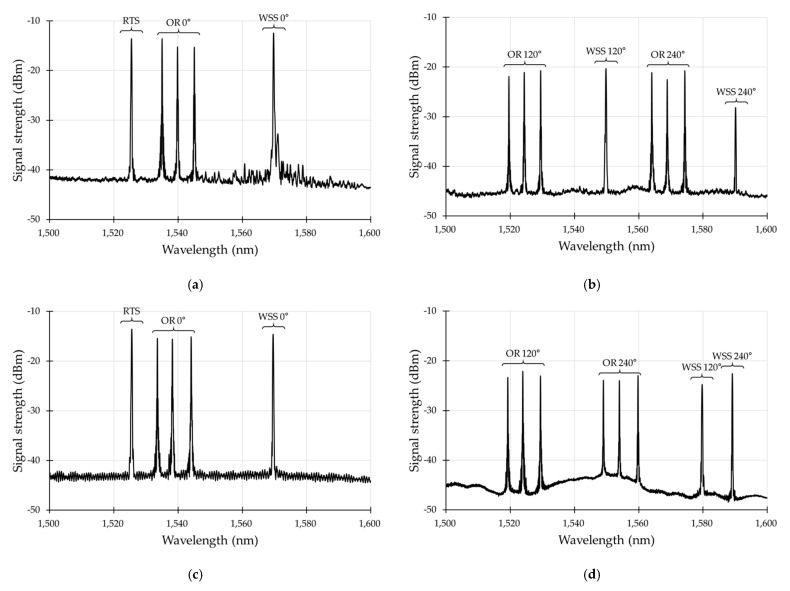
Optical spectrum of the FBG sensor after the completion of the TEBD observatory cement curing: (**a**) CH 1; (**b**) CH 2; (**c**) CH 3; (**d**) CH 4.

The sensors were successfully installed at two observatories after applying several improvements through trial and error. Figure 15 shows the optical spectrum results from the FBG sensor installed at depths of 500 m and 1000 m at the TEBD observation station. We could identify that all sensors worked well with satisfactory SNR level greater than 20 dB.

## 4. Discussion

The findings and learnings obtained from the above-mentioned two cases can be summarized as follows:

1. Sensor selection: To secure the sensor performance and long-term attachment stability of the sensor, weldable-type sensors, if available, are preferable. In the case described above, the bonding-type sensor was used because the radius of curvature of the casing was not >300 mm, which is the minimum radius of curvature acceptable for the 3-component weldable strain sensor;
2. FRP forming method: The bagging method, regarded as superior to the hand lay-up method for sensor protection, removes the excessive epoxy, and thus, the volume of the sensor part is reduced, and the sensors and cables are more uniformly packaged. During the process of sucking out the epoxy resin, however, air infiltrated into the package or residual air bubbles within the epoxy resin may form unwanted inflow channels of groundwater. Infiltrated ground water may deform intermediate protection materials like AK22 and ABM75 to cause excessive bending or cutting of optical fiber. When the bagging method is used, close attention should be paid to additional waterproofing and air removal measures during the excessive epoxy removal process;
3. Glass fiber: Several lamination layers of the glass fiber are preferred considering the limited drilling hole conditions. Following previous studies [41,42,43], using two glass fibers with different structures during this process looks effective in improving the water tightness, interlayer adhesivity, and tensile/impulse strength;
4. Epoxy and hardener: The proper selection of the hardener depends on the temperature and time. With a faster hardener, more heat is generated during curing, and excessive overheating can cause damage to the inner optical fiber. Therefore, the use of a slower hardener is recommended;
5. Optical fiber: In the composition of the optical fiber of the fiber cable, two MMFs are sufficient, even considering the dual-ended configuration for the DTS measurement. Since manufacturing cost is not significantly affected by the number of SMFs, including as many SMFs is recommended as long as the cable specification and technology allows in order to increase the degree of freedom of the serial/parallel configuration of the FBG sensor and wavelength division;
6. Optical fiber cable: The tube-in-tube structure can lead to the slip phenomenon, which can be prevented by installing a reducing union on the optical fiber cable. In less harsh and tough environments, using a single tube can also be considered as an alternative;
7. Optical fiber fusion connection component: A protection method using ABM75 and FRP in the covering compound shape is effective. When using ABM75, the space to arrange the optical fiber and optical fiber fusion splice is secured, and the work can be easily performed, further reducing the working hours needed.

## 5. Conclusions

In this study, we investigated the installation and protection methodology for using an optical fiber sensor in borehole environments through two cases of installing FBG strain sensors inside deep boreholes. 

When using an optical fiber sensor for long-term monitoring in a deep borehole environment, the main requirements are to (1) secure the optimal performance and long-term durability of the sensor under the physicochemical conditions of the sensor location, and (2) prevent the loss or damage of the sensor and cable in the process of installing them into the narrow and deep space.

Optical fiber sensors are highly suitable for use in the earth science field because they offer advantages such as immunity against any form of electrical interference, durability, and cost-effectiveness. Nevertheless, studies on additional measures to protect the optical fiber sensors, cable, and connections between them are still needed to increase the field applicability of the optical fiber sensor in geoscientific fields. In addition, it is also expected that the development of more advanced fiber-optical sensor and packaging technology optimized for the harsh environment of the geoscience field will contribute to expanding applicability.

## Figures and Tables

**Table 1:** FBG sensor specifications.

Product Name	OR	WSS	RTS	FBG AC
Manufacturer	HBM	HBM	HBM	FBG Korea
Measurement parameter	3-Axis Strain	1-Axis Strain	Temperature	2-Axis Acceleration
Sensitivity	1.2 pm/με	1.2 pm/με	30 pm/°C	600 pm/G
Measurement range	±10,000 με	±5000 με	−20 °C~80 °C	±2 G
FBG reflectivity	<15%	>65%	>65%	>70%
Full width at half maximum (FWHM)	-	>0.2 nm	>0.2 nm	≤0.3 nm
Fiber core and cladding diameter	6/125 μm	8/125 μm	8/125 μm	-
Attachment method	Bonding	Spot Weld	Directly Cast	Arc Welding
Operating temperature range	−10 °C~80 °C	−20 °C~80 °C	−20 °C~80 °C	−20 °C~80 °C
Minimum bend radius	25 mm	400 mm	Cannot Bend	Cannot Bend
Dimensions	42.7 × 46.8 × 2.0 mm (L × W × T)	83 × 23 × 6 mm (L × W × T)	100 × 10 mm (L × Ø)	90 × 37 × 36 mm (L × W × H)
